# Expeditious Quantification of Lignocellulolytic Enzymes from Indigenous Wood Rot and Litter Degrading Fungi from Tropical Dry Evergreen Forests of Tamil Nadu

**DOI:** 10.1155/2014/127848

**Published:** 2014-02-26

**Authors:** Jenefar Sudarson, Shenbhagaraman Ramalingam, Premalatha Kishorekumar, Kaviyarasan Venkatesan

**Affiliations:** ^1^Mycology Laboratory, Centre for Advanced Studies in Botany, University of Madras, Chennai 600 025, India; ^2^Centre for Nanoscience and Technology, Anna University, Chennai 600 025, India

## Abstract

In this study thirty wood rotting and litter degrading basidiomycetes were screened for the production of lignocellulolytic enzymes such as, laccase, peroxidase, and cellulase using rapid micro quantification assay. Out of the 30 indigenous isolates *Trametes gibbosa* was identified to be a potential lignocellulolytic enzyme producer, producing a maximum amount of cellulase (299.66 ± 1.59 IU/L) and laccase (257.94 ± 1.79 U/L). Moreover, it is the second leading producer of peroxidase enzyme (170.19 ± 1.98 U/L). *Tricholomopsis* sp. a wood rot basidiomycete was found to be the leading lignin decomposer with maximum peroxidase activity (287.84 ± 2 U/L) and second maximum laccase activity (250.19 ± 1.83 U/L). However, its cellulolytic potential was found to be moderate (100.04 ± 1.13 U/L). A higher level of lignocellulolytic enzymes was recorded in wood rotting basidiomycetes, whereas very low levels of lignolytic enzymes were found in litter inhabiting basidiomycetes. However, their cellulolytic potential was found to be moderate.

## 1. Introduction

Lignocellulosic substrates have recently gained remarkable interest due to their wide biotechnological applications in the agricultural industry, food processing, paper, and fuel industries. The biotechnological process not only uses lignocellulosic wastes as an energy feedstock but is also associated with pollution abatement [1, 2]. These substrates are mainly composed of cellulose, hemicellulose, and lignin [3]. Cellulose is a biopolymer and has been widely used in paper making, as a source of sugars, and as a precursor for bioethanol production and for various purposes. Recovery of cellulose from lignocellulosic substrates of physical and chemical methods is an energy intensive process as the lignin acts as barrier for them [4]. Naturally, the cellulose from these lignocellulosic substrates can be utilized by a wide variety of wood rotting and litter degrading fungi. They produce enzymes such as laccase and peroxidase for the degradation of lignin and cellulase for the cellulose utilization. In general, mushrooms become accustomed to the abundant supply of lignocellulosic substrates, digest them, utilize them for their growth, and thereby they recycle them. The efficiency of utilizing lignocellulosic waste materials depends on their ability to secrete potential hydrolytic, oxidative enzymes which differ from species to species [5]. Studies have shown that wood rot fungi invest part of their metabolic energy to produce lignocellulolytic enzymes for the purpose of degrading lignin [6, 7]. The enzymes produced using agroindustrial or organic wastes from mushrooms have wide application in the field of diagnostic medicine, textile, paper, and biofuel industries, which accounts for 40% of global enzyme market supply [8–10].

The major objective of this study is to tap out the potential lignocellulolytic enzyme producer from various wood rot and litter fungi and to compare them. Screening is the major strategy to identify the efficient industrially viable enzyme producer from environmental sources. Efficient, rapid screening systems are needed to identify and quantify these classes of enzymes using specific substrate. Hence, in this study a rapid microquantification assay has been used for the determination of lignocellulolytic enzymes such as laccase, peroxidase, and cellulase from wood rot and litter degrading basidiomycetes collected indigenously from places in and around Chennai.

## 2. Materials and Methods

### 2.1. Chemicals

2,2′-Azino-bis-3-ethylbenzothiazoline-6-sulfonate (ABTS), 2-methoxyphenol, and carboxymethyl cellulose were from Sigma-Aldrich, and H_2_O_2_ (perhydrol, 30%) was obtained from Boehringer. All other chemicals used were of analytical grade.

### 2.2. Organisms and Fermentation for Enzyme Production

The fruiting bodies of mushrooms found growing on the trees, decomposing logs, and soils at different places in and around Chennai, Tamil Nadu, India, were collected and isolated in pure cultures on PDA and revived before every assay. The collected mushrooms were further identified using field characters such as substrate for growth and occurrence of fruiting body [11], morphological characters such as pileus, lamellae, stipe, and fruiting bodies [12], and microscopic characters such as spore print, cystidia, and hyphal arrangement [13]. The isolated culture was deposited in the fungal culture collection, Centre for Advanced Studies in Botany. The fungal isolates were precultured in Potato dextrose agar (PDA) medium at 28°C for 14 days and agar plugs (10 mm in diameter) were inoculated in the liquid medium. A basal liquid medium was prepared in the following composition: glucose—10 g/L, yeast extract—3 g/L, peptone—1 g/L, MgSO_4_·7H_2_O—1 g/L, and KH_2_PO_4_·3H_2_O—1 g/L. The mycelium from the plate was inoculated with 50 mL of liquid medium in a 250 mL Erlenmeyer flask. The cultures were incubated at 25°C for 30 days under static condition at optimum pH of 6.5 ± 0.5. The culture filtrate was then separated and centrifuged at 5000 rpm for 15 min. The supernatant was carefully transferred and was treated as the crude enzyme fluid and assays were performed by the microtitre plate method.

### 2.3. Microquantification of Lignocellulolytic Enzymes

The laccase activity was monitored by measuring the maximum absorption of oxidation of ABTS (2,2′-azinobis-3-ethylbenzthiazoline-6-sulfonate) substrate at 25°C. The reaction mixture (200 *μ*L) containing 10 *μ*L of enzyme sample, 10 *μ*L of 10 mM/L ABTS, and 180 *μ*L of 50 mM/L sodium acetate buffer solution (pH of 4.5) was incubated for 3 min and the laccase activity of crude enzyme was determined by measuring the absorbance at 420 nm using ELISA Reader model Multiskan EX. One unit of enzyme activity was defined as the amount of enzyme catalyzing the oxidation of 1 *μ*mol of substrate per minute [14].

Peroxidase activity was determined by monitoring the oxidation of guaiacol at room temperature, that is, 25 ± 2°C. The reaction mixture (200 *μ*L) contained 100 mM of citrate phosphate buffer (pH 4.0), 1 mM of 30% hydrogen peroxidase solution, 1 mM of guaiacol, and the supernatant of culture filtrate. The absorbance was determined at 414 nm using ELISA reader model Multiskan EX. One unit of peroxidase activity was defined as the amount of the enzyme, which leads to the oxidation of 1 *μ*mol of substrate per minute [15]. Both the laccase and total peroxidase were performed in microtitre plates [16, 17].

Cellulase was initially done with tubes, then the final incubation and the absorbance were carried out in microtitre plates. Cellulase activity was assayed by mixing 50 *μ*L of proper enzyme dilution with 50 *μ*L of 2% carboxymethylcellulose solution in a 0.05 M citrate buffer (pH 4.8) and incubating the mixture for 30 min at 50°C in a water bath with moderate shaking. Dinitrosalicylic acid was added and boiled for 5 min. The absorbance was measured at 540 nm [18]. The sample and buffer were poured into the well using multichannel pipettes. All the samples were measured with microtitre plate reader model Multiskan EX [19]. One unit of CMC activity is defined as the amount of enzyme needed to liberate 1 mol of glucose/min. Glucose was used as standard for CMC activity.

### 2.4. Statistical Analysis

Values are expressed as means ± S.D. and analyzed using one-way ANOVA for comparisons of means. The statistical analysis was performed using SPSS version 10 for Windows (SPSS, Inc.).

## 3. Results 

**Figure 1:**
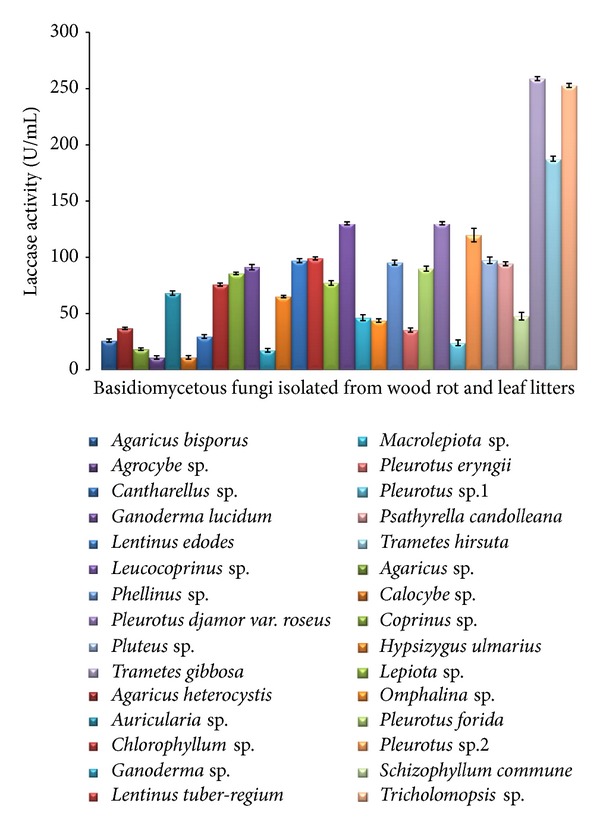
Quantification of Laccase activity determined using ABTS by microtitre plate assay. Extracellular enzymes of laccase production from cultures of basidiomycetes. All values are medium of three replications ± standard error.

**Figure 2:**
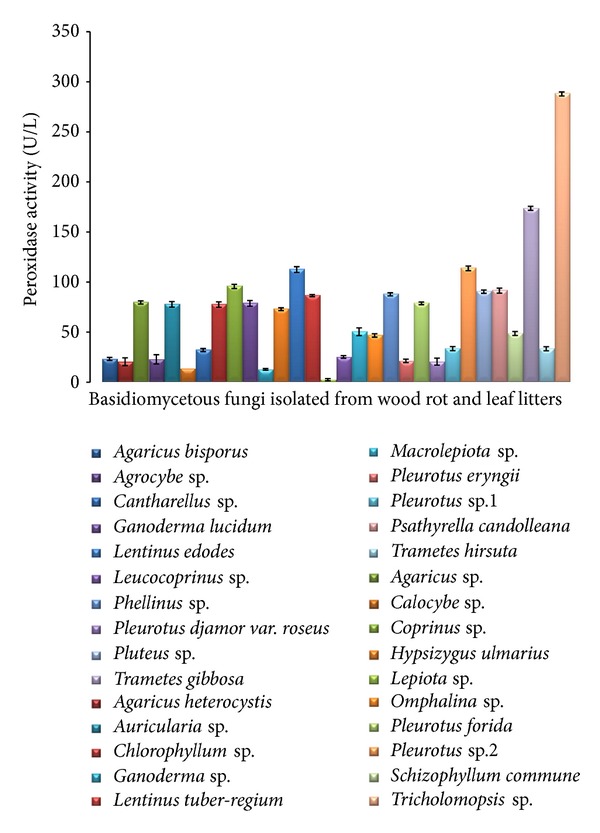
Quantification of peroxidase activity using guaiacol by microtitre plate assay. Extracellular enzymes of peroxidase production from cultures of basidiomycetes. All values are medium of three replications ± standard error.

A total of 30 indigenous collected species of basidiomycetes were identified and substrates from which it is isolated were mentioned in Table 1. The collected basidiomycetes were evaluated for their extracellular lignocellulolytic enzyme production using a microtitre plate method after submerged fermentation. Out of these enzymes, laccase plays a dynamic role as the best lignin upgrade in lignin degrading fungi. The results of the screening of laccase were shown in Figure 1; it shows that *Trametes gibbosa* (257.94 ± 1.79 U/L) was the highest producer of this enzyme, which was followed by *Tricholomopsis *sp. (250.19 ± 1.83 U/L) and *Trametes hirsuta* (185.95 ± 2.33 U/L), whereas a low enzyme activity was observed in *Agrocybe* sp. (13.23 ± 1.45 U/L) (Figure 2).

**Figure 3:**
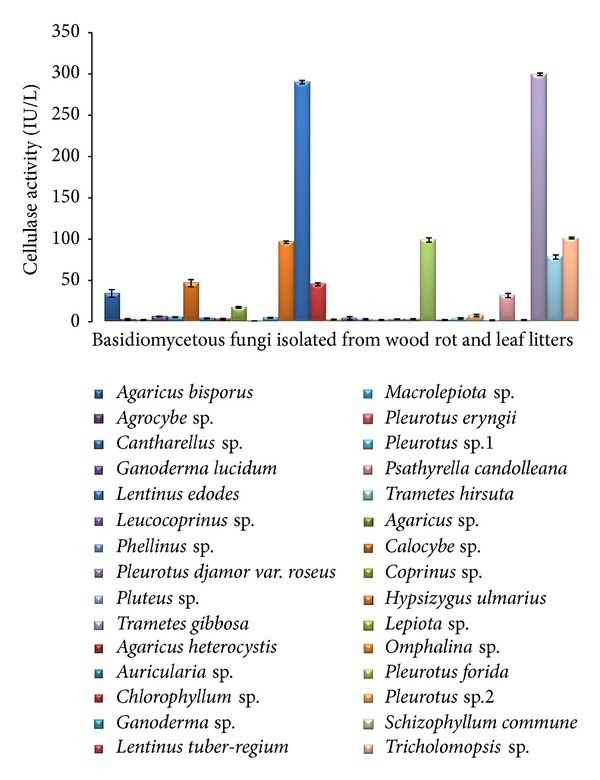
Quantification of cellulase activity using carboxymethyl cellulose by microtitre plate assay. Extracellular enzymes of cellulase production from cultures of basidiomycetes. All values are medium of three replications ± standard error.

Peroxidases are one of the key enzymes responsible for the degradation of lignocellulose, of which peroxidases are considered to be the most effective in the removal of lignin and were quantified in this study using rapid microtitre plate based quantitative peroxidase assay (Figure 3). In this study, *Tricholomopsis* sp. showed the highest peroxidase activity of 287.84 ± 2 U/L followed by *Trametes gibbosa* (170.19 ± 1.98 U/L) and *Lentinus edodes* (117.96 ± 2.88 U/L) (Figure 2). Very least peroxidase activity was recorded in *Lepiota* sp. (4.7 ± 1.11 U/L) (Figure 2).

Cellulase refers to hydrolytic enzymes that catalyse the cellulolysis. Cellulase have wide range of potential applications in various industries. Figure 3 revealed that, out of 30 fungi, six indigenous fungi were identified with a potential cellulolytic capacity. *Trametes gibbosa*, member of the polyporus fungi, exhibited the highest cellulolytic activity of 299.143 ± 1.59 IU/L (Figure 3). The other wood rots *Lentinus edodes* and *Tricholomopsis* sp. Were recorded to possess the highest enzyme activity (294.143 ± 2.08 IU/L and 101.044 ± 1.13 IU/L, resp.). However, *Hypsizygus ulmarius, Pleurotus florida*, and *Tramates hirsuta* were also shown to possess considerable cellulolytic activity of 98.42 ± 1.45 IU/L, 93.78 ± 2.6 IU/L, and 79.23 ± 2.55 IU/L, respectively.

## 4. Discussion

A number of research works were carried out to evaluate the enzymatic potential by classical methods and these methods are time consuming and require more amount of substrate for quantification. Hence, to quantify the lignocellulolytic enzymes in short duration, microquantification technique is the best method of choice. Most wood inhabiting fungi showed good laccase activity except a few species such as *Ganoderma* sp., *Calocybe *sp., and one wild *Pleurotus *sp. where very low level of enzyme activity was recorded. Conversely, the litter inhabiting fungi such as *Agrocybe *sp. and* Agaricus *sp. showed very low level of enzyme activity. This is due to the difference in substrate in which they grow; that is, the wood rot fungi produce more laccase enzyme than the litter degrading fungi [20]. The laccase enzyme finds its major application in processes such as delignification, biopulping, biosorption, and wine clarification, and *Trametes *was the first reported laccase producer. Out of the different cultures screened *Trametes gibbosa* and *Trametes hirsuta* have been proven to be potential candidates with the highest laccase activity, in addition to *Tricholomopsis *sp. Thus, the results of our study correlate with studies made by Songulashvili et al. [21] where the study showed that the genus *Trametes* expressed comparatively a higher laccase activity than the other species of wood rot basidiomycetes. Moreover, most common laccase producers are wood rot fungi; especially, polyporales play a major role in efficient degradation of lignin [20, 22]. Similarly, in this study most of white rots including *Lentinus edodes*, *Pleurotus djamor var. roseus,* and* Pleurotus* sp. 2 cultures exhibited a comparatively better laccase activity. Screening methods play a major role in the identification of potential candidate for biotechnological applications. The rapid microtitre plate screening method used in this study showed promising results similar to those of Okino et al. [23] where they developed a quick screening method and isolated 116 Brazilian tropical rainforest basidiomycetes expressing laccase enzyme. Substrate for the enzyme is another factor for accurate quantification of the enzymes. In case of enzyme laccase, ABTS was found to be the suitable substrate, as it rapidly detects this enzyme more accurately in this method.

For identification of a high level peroxidase producers, the time and reliability are the considerable factors for determining the activity. Rapid microquantification assay was proved to be the reliable and short time method for determination of peroxidase enzymes. In this study, the peroxidase enzymes were determined by rapid microquantification assay. Wood rot fungi showed higher production of extracellular peroxidase than the litter degrading basidiomycetes. This may due to the fact that wood rots require peroxidase enzyme and possess high oxidative ability to degrade lignin [24]. The rapid microtitre plate based quantitative peroxidase assay used in this study showed promising results, comparable to those of classical quantitative spectrophotometric assay based screening studies carried out by Dhouib et al. [25]; Taboada-Puig et al. [26]; Järvinen et al. [27]. Sometimes, the fungal strains from white rot group are able to produce laccase more than the peroxidase. Hence, it is essential to quantify the target catalyst from large number of strains which is extremely important. Accuracy for oxidative enzyme screening depends on the chromogenic substances used for its detection. In this study, the guaiacol was used as the chromogenic substrate for the detection of peroxidase. Similar studies by Mercer et al. [28] screened the peroxidase activity of actinomycetes using rapid microquantitative assay and demonstrated that this technique was effective in rapid screening. Out of the 30 fungal strains screened, almost all wood rot fungi exhibited significant peroxidase activity except for a few species such as *Pleurotus eryngii* and *Lepiota* sp.

Nowadays, significant attention has been devoted to the knowledge of cellulase production and the challenges in cellulase research especially in improving the process economics of various industries [29]. Cellulase had a series of applications in food, pulp, fuel, textile, and so forth. Hence, the screening of cellulolytic potetial fungi for its ability towards industry level is essential nowadays. The microquantification cellulase assay using carboxymethyl cellulose (CMC) showed promising results in this study and was comparable with that of classical screening assay in many wood rots and litter fungi tested by Dhouib et al. [25] where they screened 224 fungal strains from Zimbabwe for cellulolytic activities. The microquantification cellulase assay using CMC was the best method; evidence from previous studies was made by king et al. [30] where the study compared the cellulase production of different fungi such as *Trichoderma reesei, Fusarium oxysporum, and S. sclerotina, *using microtitre plate methods. The results showed that *T. reesei* showed maximum cellulase production in CMC and arabinoxylan substrates. Wang et al. [31] reported that three strains of *Agrocybe aegerita* utilized non-lignin-cellulose more efficiently than the other strains in the study and their cellulose-degrading activity was slightly lower. Similar results were observed in our study that *Agrocybe *sp. exhibited lower cellulase activity. White rot fungi produced more laccase and peroxidase extracellularly than cellulase at low rate during anamorphic phase of the basidiomycetes. In contrast, the *Trametes* produces cellulase at high levels than the litter degrading fungi, which may due to the factors such as diversity of the environment, adaption, evolution, and modification of genes by the organisms [32, 33].

The rapid quantification assay technique used in this screening study identified the efficient lignocellulolytic enzymes producing indigenous isolates such as *Trametes gibbosa, Tricholomopsis *sp*., Trametes hirsuta, Lentinus edodes,* and *Pleurotus* species from native environments of Tamil Nadu, India. Nevertheless, most of the white rots and litter degrading fungi produce laccase, peroxidase and cellulase enzymes, their level differs depends on the substrate that is wood or soil or litter, from which it is isolated. Some of the same genus have different level of enzyme production which is totally based on the species variation and also the genetic modification of the strains. Thus, the study emphasis to explore the basidiomycetes fungi and its oxidative and hydrolytic enzymes was to evaluate the accurate efficacy of the fungi.

## 5. Conclusion

Thirty south Indian taxa of basidiomycetes were collected and screened for extracellular oxidative and hydrolytic enzymes using microtitre plate technique. The most promising results obtained with these ligninolytic fungal strains led to discovering the hidden potentials of some of the members of basidiomycetes. This study strives to unravel the immense lignin-degrading potential of basidiomycetes from South India and also to make this data available to promote future research.

## Figures and Tables

**Table 1:** Fungal cultures isolated and their substrates.

Serial number	Name of organism isolated	Name of substrate	Group
1	*Agaricus bisporus *	Soil	Litter degrading basidiomycetes
2	*Agaricus heterocystis *	Soil	
3	*Agaricus *sp.	Soil	
4	*Agrocybe* sp.	Soil	
5	*Cantharellus* sp.	Soil	
6	*Chlorophyllum *sp.	Soil	
7	*Coprinus *sp.	Soil	
8	*Lepiota *sp*. *	Soil	
9	*Leucocoprinus* sp*. *	Soil	
10	*Macrolepiota *sp*. *	Soil	
11	*Omphalina *sp*. *	Soil	

12	*Auricularia *sp.	Wood	Wood rot basidiomycetes
13	*Calocybe *sp.	Wood	
14	*Ganoderma lucidum *	Wood	
15	*Ganoderma *sp*. *	Wood	
16	*Hypsizygus ulmarius *	Wood	
17	*Lentinus edodes *	Wood	
18	*Lentinus tuber-regium *	Wood	
19	*Phellinus *sp*. *	Wood	
20	*Pleurotus eryngii *	Wood	
21	*Pleurotus florida *	Wood	
22	*Pleurotus djamor var. roseus *	Wood	
23	*Pleurotus *sp.1	Wood	
24	*Pleurotus *sp.2	Wood	
25	*Pluteus* sp*. *	Wood	
26	*Psathyrella candolleana *	Wood	
27	*Schizophyllum commune *	Wood	
28	*Trametes gibbosa *	Wood	
29	*Trametes hirsuta *	Wood	
30	*Tricholomopsis *sp.	Wood	
